# Inflammation and hypertension development: A longitudinal analysis of the African-PREDICT study

**DOI:** 10.1016/j.ijchy.2020.100067

**Published:** 2020-11-21

**Authors:** Simone H. Crouch, Shani Botha-Le Roux, Christian Delles, Lesley A. Graham, Aletta E. Schutte

**Affiliations:** a Hypertension in Africa Research Team (HART), North-West University, Potchefstroom, South Africa; b MRC Research Unit: Hypertension and Cardiovascular Disease, North-West University, Potchefstroom, South Africa; c The British Heart Foundation Centre of Excellence, Institute of Cardiovascular and Medical Sciences, College of Medical, Veterinary, and Life Sciences, University of Glasgow, Glasgow, United Kingdom; d School of Population Health, University of New South Wales; The George Institute for Global Health, Sydney, Australia

**Keywords:** Hypertension, Cytokine, Ethnicity, African, Black

## Abstract

**Background:**

The role of inflammation in the development of hypertension remains incompletely understood. While single inflammatory mediators have been shown to associate with changes in blood pressure (ΔBP), the role of clusters of inflammatory mediators has been less comprehensively explored. We therefore determined whether individual or clusters of inflammatory mediators from a large biomarker panel were associated with ΔBP over 4.5 years, in young healthy adults.

**Methods:**

We included 358 adults (white, n = 156; black, n = 202) with detailed information on ambulatory blood pressure (BP) at baseline and follow-up. Baseline blood samples were analysed for 22 inflammatory mediators using multiplexing technology. Principal component analysis was used to study associations between clusters of inflammatory mediators and ΔBP.

**Results:**

In the total cohort in multivariable-adjusted regression analyses, percentage change in 24hr systolic BP associated positively with Factors 1 (Interferon-gamma, interleukin (IL)-4, IL-7, IL-10, IL-12, IL-17A, IL-21, IL-23, macrophage inflammatory protein (MIP)-1α, MIP-1β, TNF-α, granulocyte-macrophage colony-stimulating factor (GM-CSF)) and 2 (IL-5, IL-6, IL-8, IL-13). Change in daytime systolic BP associated positively with Factors 1, 2 and 3 (C-Reactive protein, IL-1β, IL-2, MIP-3α). Subgroup analysis found these findings were limited to white study participants. Numerous associations were present between individual inflammatory mediators (Interferon-gamma, GM-CSF, IL-4, IL-6, IL-7, IL-8, IL-10, IL-12, IL-13, IL-17A, IL-21, IL-23, MIP-1α and MIP-1β) and ΔBP in the white but not black subgroups.

**Conclusion:**

We found independent relationships between numerous inflammatory mediators (individual and clusters) and ΔBP over 4.5 years. The relationship between inflammatory markers and ΔBP was only found in white participants. ClinicalTrials.gov (Identifier: NCT03292094)..

## 1 Introduction

Hypertension is the most prominent risk factor for the development of cardiovascular disease [1]. The Global Burden of Disease study found raised systolic blood pressure (BP) to account for 10.4 million deaths per year [2]. Hypertension is a multi-factorial trait that develops as a result of both environmental and genetic factors [3]. One important factor found to contribute to BP elevation is low-grade inflammation [4].

The infiltration of innate and adaptive immune cells, along with other inflammatory processes such as expression of adhesion molecules, cytokines and reactive oxygen species by the brain, kidneys and the vasculature are consistently found in individuals with hypertension [[5], [6], [7], [8]]. C-reactive protein (CRP), interleukin (IL) 6, and tumour necrosis factor alpha (TNF-α) relate positively to hypertension [9], and risk prediction models that include CRP have been developed [10]. However, the inflammatory pathways involved in early vascular ageing and hypertension development may be more complex and cannot be fully represented by single markers [11]. While single inflammatory mediators have been found to be associated with raised BP [12], the role of clusters of inflammatory mediators has been less comprehensively explored. The activation of pro- and anti-inflammatory pathways does not occur in isolation and numerous interactions between inflammatory mediators exist, making investigations into the role of inflammation in the development of hypertension challenging [13].

We have shown, using data from several studies including a cross-sectional analysis in 1189 participants from the African-PREDICT study [14], that the profiles of inflammatory mediators differ in populations from African descent when compared to populations of European descent [15]. Black populations are known to have a greater burden of hypertension than most other populations [16]. As such, we hypothesised that pro-inflammatory mediators would associate adversely with change in blood pressure (ΔBP). Therefore, we evaluated the role of inflammatory mediators in the early stages of development of hypertension in both young black and white adults.

## 2 Methodology

### 2.1 Study population

**Fig. 1:**
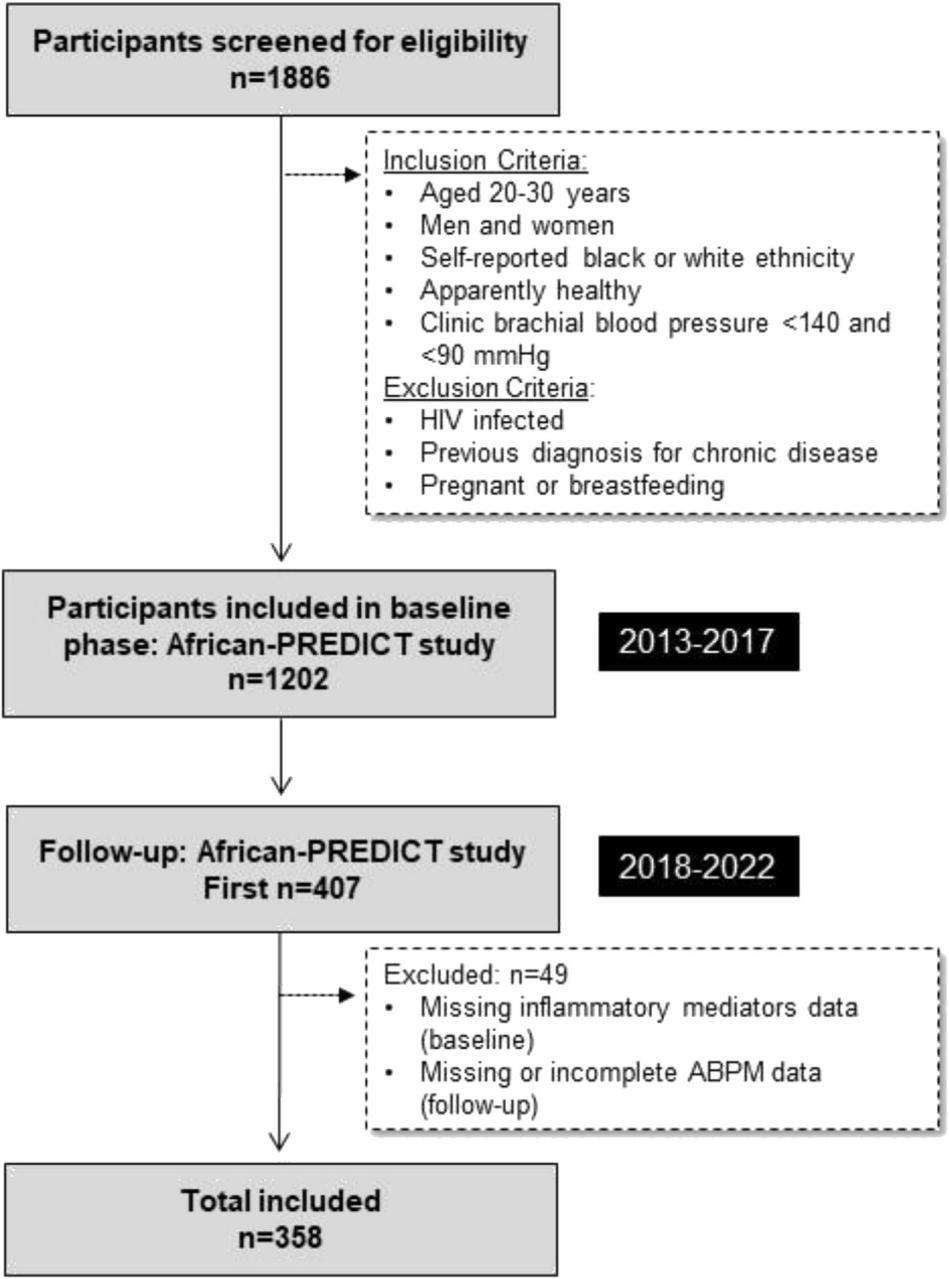
Layout of the study population.

This study forms part of the African Prospective study on the Early Detection and Identification of Cardiovascular disease and Hypertension (African-PREDICT), and detailed methods have been described before [17]. As shown in Fig. 1, from 2013 to 2017 we recruited 1202 young black and white men and women, between the ages of 20–30 years. Participants were recruited from Potchefstroom and surrounding areas in the North West Province, South Africa via community health workers, workplace and public advertisement. Individuals from low, middle, and high socio-economic status groups were specifically included. Although individuals with office brachial BP of ≥140 and/or ≥90 were excluded during baseline screening, there was an average two-week period between the screening and research phases. Some participants were classified as being hypertensive based on 24hr ambulatory blood pressure during the research phase and were included in this study. This sub-study included data of the first 358 participants who were successfully followed up during 2018–2019. The study was approved by the Health Research Ethics Committee (HREC) of the North-West University (NWU-00058-18-A1), adheres to the guidelines as set out by the Declaration of Helsinki and all participants in the study provided written informed consent prior to participation.

### 2.2 General measurements

Self-reported data with regards to demographic, lifestyle, socio-economic status, and medication use were collected using questionnaires. Anthropometry was measured using standard methods [17]. Body mass index (BMI) was calculated as weight (kg)/height (m)^2^.

Socio-economic status was calculated using a point system adapted to the South African context from the Kuppuswamy's Socioeconomic Status Scale 2010 [18]. Participants were scored on education, household income and skill level. The South African Standard Classification of Occupation (SASCO) was used to classify skill level.

### 2.3 Blood pressure

All BP measurements were performed by a trained researcher using an appropriately sized cuff for each participant. Cuffs were selected from adults’ sizes and sizing was checked upon fitting.

#### 2.3.1 Office blood pressure

After a 10-min rest, duplicate brachial BP measurements were done on the left and right arms, with a 5-min interval in-between, whilst participants were seated (Dinamap® Procare 200; GE Medical Systems, Milwaukee, WI, USA). BP were measured in a temperature-controlled room in the research clinic, with a single researcher present. Hypertension using office BP was defined as SBP ≥140 mmHg and/or DBP ≥90.

#### 2.3.2 Ambulatory blood pressure

Participants non-dominant arm were fitted with a validated 24hr ambulatory BP (ABPM) monitor (Card(X)plore® CE120, Meditech, Budapest, Hungary) at approximately the same time every day (late morning). The monitor was programmed to record every 30 min during the day (06h00 to 22h00) and every hour during the night (22h00 to 06h00) [19]. The monitor was fitted in a temperature-controlled room in the research clinic. Participants were instructed on how and when to remove the ABPM monitor the following day. Participants were provided a diary card to record all activity during monitoring. Data were checked for missed measurements or premature removal. In this study, participants had a mean successful inflation rate of 83%. Participants were classified as hypertensive if they met any of the following three criteria: 24hr ABPM of SBP ≥130 mmHg and/or DBP ≥80 mmHg, a daytime ABPM of SBP ≥135 mmHg and/or DBP ≥85 mmHg, or a nighttime ABPM of SBP ≥120 mmHg and/or DBP ≥70 mmHg.

As ABPM were measured during baseline and follow-up, we calculated ΔBP over time, expressed at percentage change.

### 2.4 Biological sampling and biochemical analyses

Participants fasted overnight for at least 8hr prior to attending the day of research measurements. Blood samples were collected from the median cubital vein. The samples were prepared according to standardised protocols and stored at −80 °C until analysis.

Serum samples were analysed for high-sensitivity CRP, total cholesterol, glucose and gamma-glutamyltransferase (GGT) (Cobas Integra® 400 plus, Roche, Basel, Switzerland). Serum creatinine concentrations were measured using the Creatinine Jaffé Gen.2 reagent (Roche, Basel, Switzerland). Estimated glomerular filtration rate (eGFR) was calculated using the Chronic Kidney Disease Epidemiology (CKD-EPI) formula, without race in the equation as this is not appropriate for South African populations [20]. Serum cotinine was analysed using a chemiluminescence method on the Immulite (Siemens, Erlangen, Germany) apparatus.

A MILLIPEX Map Human High Sensitivity T Cell Magnetic Bead Panel (EMD Millipore, Merck, Missouri, USA) was used to analyse 21 inflammatory mediators from baseline samples, using Luminex xMAP technology on the Luminex 200™ analyser [14]. These include fractalkine, Granulocyte-macrophage colony-stimulating factor, Interferon-gamma, IL-1β, IL-2, IL-4, IL-5, IL-6, IL-7, IL-8, IL-10, IL-12, IL-13, IL-17A, IL-21, IL-23, Interferon-inducible T-cell alpha chemoattractant, Macrophage inflammatory protein (MIP)-1α, MIP-1β, MIP-3α and TNF-α.

### 2.5 Statistical analyses

IBM®, SPSS® version 26 (IBM Corporation, Armonk, New York) was used for data analyses. GraphPad Prism 5.03 (GraphPad Software, San Diego) was used for all graphics. Continuous variables were inspected for normality using QQ plots as well as inspection of skewness and kurtosis. Variables with non-Gaussian distributions were logarithmically transformed. Pro- to anti-inflammatory ratios were calculated based on literature [21,22], and new ratios were used based on instances where pro-inflammatory mediators were higher and anti-inflammatory mediators were lower in the black and white groups [14]. One-way and Two-way ANOVA, Chi-Square and McNemar tests were used to compare the profiles of black and white participants at baseline and follow-up. Factor analyses of the multiple inflammatory mediators were performed using the factor function of SPSS. Principal component analyses were used and factors with an eigenvalue of >1 were retained. Varimax rotation was used to obtain independent interpretable factors. A factor loading of ≥0.3 was used to interpret the factor patterns. Double loading was handled by placing the variable in the factor with the strongest loading factor. Factor scores with a cumulative percentage of >50 was subsequently used for multiple regression analyses. This procedure was followed in the total group and each ethnic group individually.

We determined the relationships between ΔBP as the dependent variable, and pro- and anti-inflammatory mediators at baseline using multivariate forward stepwise regression analyses. Variables included in forward stepwise multiple regression models were: age, sex, ethnicity, socio-economic status, waist circumference, total cholesterol, glucose, gamma-glutamyltransferase, cotinine, estimated glomerular filtration rate and activity energy expenditure. Cox-regressions were used to derive hazard ratios to determine whether inflammatory mediator factors predict the development of hypertension.

Sensitivity power analyses were performed using G∗Power 3.1 statistical analysis program [23]. This study should be able to detect an effect size of 0.0220 with a power of 80% using 358 participants as the sample size and significance level set at 0.05 for a multiple linear regression with a maximum of 11 covariates. Should participants be stratified into groups according to ethnicity, the study should be able to detect an effect size of 0.0510 with a power of 80% given a sample size of 156 and significance level set at 0.05, for a multiple linear regression with a maximum of 11 covariates.

## 3 Results

**Fig. 2:**
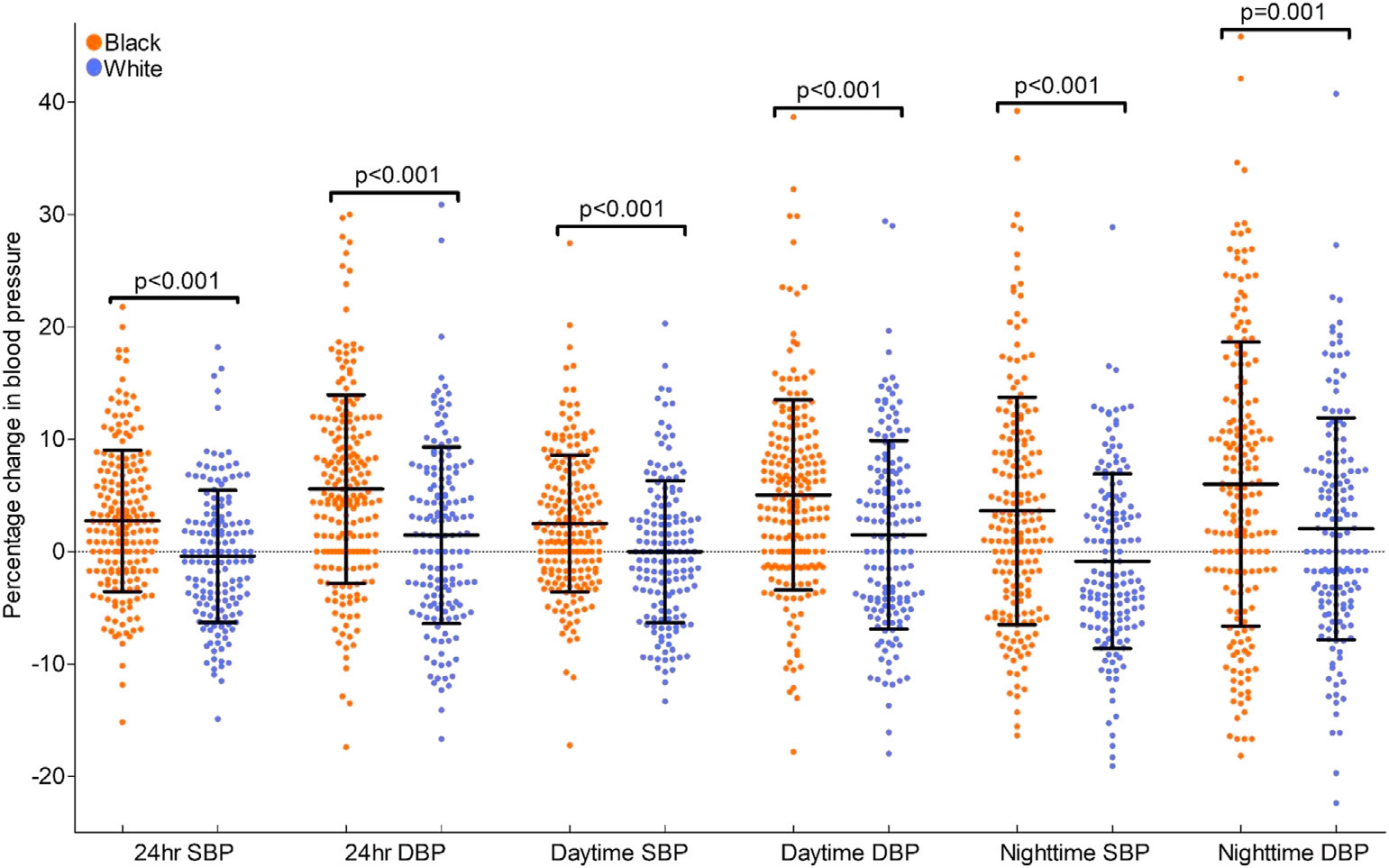
Percentage change in ambulatory blood pressure over 4.5 years in young black and white adults. Horizontal line and whiskers: Mean ± SD. Abbreviations: SBP Systolic blood pressure; DBP Diastolic blood pressure.

The general characteristics of the participants (n = 358) at baseline and follow-up are shown in Table 1. Participants had a median age increase of 4.45 years. Both groups showed an increase in BMI (p < 0.001), while an increase in waist circumference and socio-economic status were seen only in the black participants (p < 0.001). When reviewing BP, black participants showed increases in all ambulatory BP measures (all p < 0.001) during follow whereas the white participants showed an increase only in nighttime DBP (p = 0.035) (Table 1). Consequently, percental ΔBP were higher in the black compared to the white group (Fig. 2). The number of young black adults with hypertension increased from 16.8% to 42.8% over time (p < 0.001), whereas the white group showed no significant change (25.0%–33.3%, p = 0.53).

**Table 1:** Characteristics at baseline and follow-up.

	Black (n = 202)			White (n = 156)		
	Baseline	Follow-Up	p	Baseline	Follow-Up	p
Age, years	24.5 ± 3.26	28.9 ± 3.39	<0.001	26.2 ± 2.75	30.9 ± 2.70	<0.001
Men, n (%)	82 (40.6)	–		73 (46.8)	–	
**Socio-economic Status**						
Low, n (%)	129 (63.9)	74 (45.1)	<0.001	10 (6.4)	7 (5.4)	0.69
Middle, n (%)	51 (25.2)	53 (32.3)		29 (18.6)	30 (23.1)	
High, n (%)	22 (10.9)	37 (22.6)		117 (75.0)	93 (71.5)	
**Body Composition**						
Body mass index (kg/m^2^)	24.5 ± 5.45	26.8 ± 6.70	<0.001	26.4 ± 5.32	27.2 ± 5.66	<0.001
Waist circumference (cm)	76.9 ± 11.3	79.1 ± 13.6	<0.001	84.1 ± 14.7	84.8 ± 15.4	0.31
**Office BP (mmHg)**						
SBP	118 ± 12.4	117 ± 12.6	0.36	118 ± 12.3	114 ± 12.4	<0.001
DBP	79.7 ± 8.90	80.8 ± 9.82	0.078	78.4 ± 8.40	77.9 ± 8.41	0.39
**Ambulatory BP (mmHg)**						
24 h SBP	115 ± 9.01	118 ± 10.3	<0.001	119 ± 10.1	118 ± 10.8	0.27
24 h DBP	68.4 ± 5.86	72.1 ± 7.18	<0.001	69.7 ± 6.26	70.5 ± 6.84	0.054
Daytime SBP	119 ± 9.43	122 ± 10.6	<0.001	124 ± 10.7	123 ± 11.4	0.76
Daytime DBP	72.8 ± 6.44	76.3 ± 7.61	<0.001	74.7 ± 10.7	75.5 ± 7.21	0.079
Nighttime SBP	107 ± 10.3	110 ± 11.5	<0.001	109 ± 10.6	108 ± 10.7	0.072
Nighttime DBP	60.1 ± 6.94	63.5 ± 8.00	<0.001	59.8 ± 6.54	60.8 ± 7.22	0.035
Hypertensive, n (%)	34 (16.8)	86 (42.8)	<0.001	39 (25.0)	52 (33.3)	0.53

Findings presented as mean ± SD

Abbreviations: SBP Systolic blood pressure; DBP Diastolic blood pressure

When comparing the characteristics of black and white participants at baseline (Table S1), white participants were older with an overall higher socio-economic status (p < 0.001). In addition, white participants had higher BMI, waist circumference, 24hr SBP, daytime SBP and DBP, nighttime SBP and incidence of hypertension (p ≥ 0.035). The levels of most inflammatory mediators were similar (p > 0.05) between the ethnic groups at baseline. Black participants had higher levels of ITAC and MIP-3α (both p ≤ 0.006) and lower levels of IL-6, IL-10 and IL-13 (all p ≤ 0.040), similar to what we previously reported in a larger sample [14]. Additionally, black participants had higher ratios of IL-1β/IL-10, TNF-α/IL-10, MIP-1α/IL-10, ITAC/IL-4, ITAC/IL-5, ITAC/IL-10 and ITAC/IL-13 (all p ≤ 0.022).

We performed factor analyses with the pro- and anti-inflammatory mediator data to determine factor scores (Table S2-S4). Factor scores were subsequently used for multiple regression analyses to determine whether the pro- and anti-inflammatory mediator factors at baseline associate with the ΔBP. In the total population (Table 2), percentage change in 24hr SBP and daytime SBP associated positively with Factors 1 and 2, and percentage change in daytime SBP also associated positively with Factor 3. Black ethnicity contributed significantly to ΔBP in all the models. When performing these analyses separately in the two ethnic groups, in white participants (Table 2), 24hr SBP as well as 24hr daytime SBP (both p ≤ 0.020) were associated positively with Factor 1. Additionally, Factor 2 associated positively with 24hr SBP, 24hr DBP, 24hr daytime SBP and 24hr daytime DBP (all p ≤ 0.038). No statistically significant associations were found in the black group (Table 2). We then stratified white subjects according to the degree of change in 24hr SBP and DBP (Table S5). Factor 2 negatively associated with 24hr SBP in quartile 3 while Factor 3 positively associated with 24hr SBP in quartile 2 (all p ≤ 0.021). Comparison of the upper quartile with the lowest quartile revealed no significant results. This additional analysis did not define a particular subgroup at risk.

**Table 2:** Multivariable adjusted forward stepwise regression analyses in the total group to show the relationship between percentage change in blood pressure and clusters of inflammatory mediators.

Percentage change in						
**Total**	**24hr SBP (n = 354)**	**24hr DBP (n = 356)**	**Daytime SBP (n = 354)**	**Daytime DBP (n = 355)**	**Nighttime SBP (n = 340)**	**Nighttime DBP (n = 341)**
	R^2^ = 0.083	R^2^ = 0.073	R^2^ = 0.052	R^2^ = 0.039	R^2^ = 0.053	R^2^ = 0.025
Factor 1	β = 0.120	–	β = 0.142	–	–	–
	(0.008; 0.232)		(0.028; 0.255)			
	P = 0.035		P = 0.015			
Ethnicity	β = −0.182	β = −0.206	β = −0.197	β = −0.205	β = −0.238	β = −0.170
(0, black/1, white)	(−0.311; −0.055)	(−0.324; −0.091)	(−0.313; −0.084)	(−0.322; −0.092)	(−0.357; −0.123)	(−0.291; −0.052)
	P = 0.005	P = 0.001	P = 0.001	P < 0.001	P < 0.001	P = 0.005
Total Cholesterol (mmol/L)	β = −0.140	–	–	–	–	–
	(−0.289; −0.014)					
	P = 0.035					
Age (years)	–	β = −0.146 (−0.256; −0.029) P = 0.014	–	–	–	–
	R^2^ = 0.088	R^2^ = 0.073	R^2^ = 0.058	R^2^ = 0.039	R^2^ = 0.053	R^2^ = 0.025
Factor 2	β = 0.140	–	β = 0.160	–	–	–
	(0.027; 0.252)		(0.046; 0.275)			
	P = 0.035		P = 0.006			
Ethnicity	β = −0.202	β = −0.206	β = −0.218	β = −0.205	β = −0.238	β = −0.170
(0, black/1, white)	(−0.332; −0.074)	(−0.324; −0.091)	(−0.334; −0.104)	(−0.322; −0.092)	(−0.357; −0.123)	(−0.291; −0.052)
	P = 0.002	P = 0.001	P < 0.001	P < 0.001	P < 0.001	P = 0.005
Total Cholesterol (mmol/L)	β = −0.136	–	–	–	–	–
	(−0.284; −0.010)					
	P = 0.002					
Age (years)	–	β = −0.146	–	–	–	–
		(−0.256; −0.029)				
		P = 0.014				
	R^2^ = 0.072	R^2^ = 0.072	R^2^ = 0.046	R^2^ = 0.039	R^2^ = 0.053	R^2^ = 0.025
Factor 3	–	–	β = 0.118	–	–	–
			(0.002; 0.34)			
			P = 0.046			
Ethnicity	β = −0.180	β = −0.206	β = −0.193	β = −0.205	β = −0.23	β = −0.170
(0, black/1, white)	(−0.311; −0.050)	(−0.326; −0.088)	(−0.311; −0.078)	(−0.324; −0.090)	8 (-0.357; −0.123)	(−0.291; −0.052)
	P = 0.007	P = 0.001	P = 0.001	P = 0.001	P < 0.001	P = 0.005
Total Cholesterol (mmol/L)	β = −0.146	–	–	–	–	–
	(−0.297; −0.017)					
	P = 0.028					
Age (years)	–	β = −0.146	–	–	–	–
		(−0.258; −0.027)				
		P = 0.014				
**White**	**24hr SBP (n** = **156)**	**24hr DBP (n** = **156)**	**Daytime SBP (n** = **156)**	**Daytime DBP (n** = **155)**	**Nighttime SBP (n** = **154)**	**Nighttime DBP (n** = **154)**
	R^2^ = 0.032		R^2^ = 0.039			
Factor 1	β = 0.198	–	β = 0.215	–	–	–
	(0.029; 0.340)		(0.049; 0.381)			
	P = 0.020		P = 0.012			
	R^2^ = 0.033	R^2^ = 0.024	R^2^ = 0.036	R^2^ = 0.026		
Factor 2	β = 0.200	β = 0.178	β = 0.207	β = 0.183	–	–
	(0.031; 0.341)	(0.009; 0.323)	(0.040; 0.373)	(0.015; 0.342)		
	P = 0.019	P = 0.038	P = 0.015	P = 0.033		
Factor 3	–	–	–	–	–	–
Factor 4	–	–	–	–	–	–
**Black**	**24hr SBP (n** = **198)**	**24hr DBP (n** = **200)**	**Daytime SBP (n** = **198)**	**Daytime DBP (n** = **200)**	**Nighttime SBP (n** = **186)**	**Nighttime DBP (n** = **187)**
Factor 1	–	–	–	–	–	–
Factor 2	–	–	–	–	–	–
Factor 3	–	–	–	–	–	–
**Total** Factor 1: Fractalkine, IFN-γ, IL-4, IL-7, IL-10, IL-12, IL-17A, IL-23, ITAC, MIP-1α, MIP-1β, TNF-α, GM-CSF Factor 2: IL-6, IL-8, IL-13 Factor 3: IL-1β, IL-2, IL-5, IL-21, MIP-3α **White** Factor 1: IFN-γ, IL-4, IL-7, IL-10, IL-12, IL-17A, IL-21, IL-23, MIP-1α, MIP-1β, TNF-α, GM-CSF Factor 2: IL-5, IL-6, IL-8, IL-13 Factor 3: CRP, IL-1β, IL-2, MIP-3α Factor 4: Fractalkine, ITAC **Black** Factor 1: Fractalkine, IFN-γ, IL-4, IL-7, IL-10, IL-12, IL-17A, IL-23, ITAC, MIP-1α, MIP-1β, TNF-α, GM-CSF Factor 2: IL-6, IL-8, IL-13 Factor 3: IL-1β, IL-2, IL-5, IL-21, MIP-3α Findings presented as β (95%CI). Adjusted for: age, sex, socio-economic status, waist circumference, total cholesterol, glucose, gamma-glutamyltransferase, cotinine, estimated glomerular filtration rate and activity energy expenditure. Abbreviations: SBP Systolic blood pressure; DBP Diastolic blood pressure. Abbreviations: SBP Systolic blood pressure; DBP Diastolic blood pressure.						

Abbreviations: SBP Systolic blood pressure; DBP Diastolic blood pressure.

To dissect the potential contribution of individual inflammatory markers to BP changes, we also performed multiple regression analyses on individual inflammatory mediators. In white participants (Table S6), numerous associations were present between pro- and anti-inflammatory mediators (IFN-γ, GM-CSF, IL-4, IL-6, IL-7, IL-8, IL-10, IL-12, IL-13, IL-17A, IL-21, IL-23, MIP-1α and MIP-1β) and ΔBP after adjusting for multiple covariates (p < 0.05). No associations were found for black participants (Table S6).

We calculated hazard ratios (Table S7) to determine whether inflammatory mediator factors predict the development of hypertension. Factor 3 was found to predict reduced risk for hypertension (HR 0.778, 95% CI (0.611; 0.990), p = 0.042). Black ethnicity and waist circumference significantly contributed to risk prediction for all models.

## 4 Discussion

Inflammation has been implicated in the development of cardiovascular disease, including hypertension [24,25]. There is, however, a very limited understanding of whether complex inflammatory processes are already involved in the early phases of cardiovascular disease development in humans. In this study we evaluated a detailed panel of pro- and anti-inflammatory mediators in young, apparently healthy black and white adults and determined whether these mediators predict ΔBP over 4.5 years. We found independent, positive associations between clusters of inflammatory markers and ΔBP in the total group. Although black ethnicity also associated with ΔBP, associations between individual and clusters of inflammatory mediators with ΔBP were evident in white individuals only.

Other studies conducted in 6112 children aged 8–17 years [26], 193 obese children with a mean age of 13 [27], and 281 obese children aged 6–18 years [28], all found positive relationships between BP and the well-known inflammatory mediators CRP, IL-6 and IL-1β. We, however, found no associations between CRP or IL-1β and any measure of BP, but did find that IL-6 associates with ΔBP. The limited studies available examining these relationships in young individuals focussed on a restricted number of inflammatory mediators, as indicated above [[26], [27], [28]]. Our findings allowed us to show that relationships with BP are in fact present across a wide range of inflammatory mediators, further emphasising the link between inflammation and BP.

Non-steroidal anti-inflammatory drugs used to treat inflammation have been shown to raise BP as opposed to lowering it [29] – thereby demonstrating that the relationship between inflammation and BP is complex. In this study we used factor analyses to investigate the relationship between BP and clusters of inflammatory mediators. This approach may help to provide clarity on the mediators or inflammatory pathways involved. Factor 1 consisted largely of pro-inflammatory mediators, including IL-12 [30], IL-17A [6,31] and TNF-α [31], which have previously been found to associate with increased BP in rat and human models. Factor 1 also included IL-7, IL-23, MIP-1α and MIP-1β which associated positively with ΔBP. Although a study did find IL-7 to be higher in participants with hypertension than in the control group [32], research into the relationship of these mediators with BP remains limited.

Factor 2 comprised mostly of anti-inflammatory mediators such as IL-13, which despite positively correlating with BP in our study, was previously suggested to elicit a protective cardiovascular role [33]. Factor 2 additionally included IL-6 which has been associated with increased BP in previous studies [12,31], and IL-5 for which no previous reports on a direct association with BP could be found. Both Factors 1 and 2 showed robust positive relationships with ΔBP – most prominently with measures of systolic BP, in the white group. This finding supports the notion that the complex interplay between inflammatory mediators plays a role in BP regulation and not only pro-inflammatory mediators [34]. The independent associations between ΔBP and inflammatory mediators in young healthy adults suggest that inflammatory mediators may be early indicators of cardiovascular change, reflected by increasing BP.

We should acknowledge that each factor comprised of combinations of pro- and anti-inflammatory meditators, which challenges our interpretations. E.g. despite Factor 3 correlating positively with daytime SBP in the total population, it also predicted a lower risk for the development of hypertension. This may be due to the balance of pro-to-anti-inflammatory ratio and as such may highlight the potential modulatory effect a balanced inflammatory mediator profile may have on BP.

Hypertension and other cardiovascular diseases are prominent features in populations of African descent. Previous South African studies have shown that black participants display higher levels of pro-inflammatory mediators than their white counterparts [35,36]. In the larger young African-PREDICT baseline cohort (n = 1189), we also previously showed that black participants have an overall more pro-inflammatory profile than their white counterparts [14]. It is therefore surprising that the significant relationship between inflammatory mediators and ΔBP was only present in the white participants of our study. The prominent increase in BP over 4–5 years seen only in the young black population (Fig. 2) may result from factors other than their overall pro-inflammatory profile. Another study also reported an accelerated progression from prehypertension to hypertension in black compared to white counterparts [37]. When examining potential driving factors for the increase in BP seen in black participants, it is important to note that black participants showed an increase in socio-economic status, waist circumference and BMI over the follow-up period. It is well established that adiposity associates with increases in BP via numerous mechanisms [38]. While overall the white participants still showed a higher BMI and waist circumference, the shift seen in black participants from a normal BMI to an overweight BMI and the increase in waist circumference may contribute to the increase in BP seen in this group. In addition, the increase in overall socio-economic status seen in black participants may be associated with lifestyle and dietary changes, potentially contributing to an increase in BP [39]. Further driving factors may include genetic polymorphisms in renal sodium handling resulting in salt-sensitivity and low plasma renin levels [40]. Additionally, even at younger ages black populations have shown to have increased arterial stiffness [41], vascular resistance [42,43], and left ventricular mass [44] all of which may drive increases in BP [45]. In the white group, subtle changes in inflammation appear to be associated with more subtle ΔBP and it is unclear how much inflammation will contribute to hypertension in the longer term.

It is well established that biological age and chronological age are not necessarily comparable [46]. A study found that the progression of vascular ageing is accelerated in the presence of cardiovascular risk factors [47]. Exposure to risk factors in early life plays a prominent role in the deterioration of vascular structure and function [47]. It was suggested that childhood environment could have an effect on the development of inflammatory phenotypes [48], suggesting that the effects of the inflammatory mediator profile on the cardiovascular system may start at a very early age. Studies into the mechanisms involved have shown that both innate and adaptive immune responses contribute to the pathophysiology of hypertension via inflammatory changes in the kidney, blood vessels and the brain [6]. Inflammatory mediators aid in the development of hypertension through contribution to increased vascular permeability, release of cytokines, reactive oxygen species, nitric oxide and metalloproteinases [49]. Cytokine release leads to decreased lumen diameter of resistance vessels, increased vascular resistance and stiffness [49], angiotensinogen and angiotensin II production, as well as sodium and volume retention [6].

A strength of this study is the large panel of pro- and anti-inflammatory mediators which was analysed with a high-sensitivity analysis kit. The use of factor analyses allowed to identify clusters of multiple inflammatory mediators. We included young adults, which allowed us to examine the relationship between inflammation and BP in the absence of pre-existing health conditions. Furthermore, the presence of individuals with early hypertension ensures relationships are not confounded by target organ damage. However, due to the young healthy status of the cohort, it is unlikely that inflammatory mediators would predict clinical hypertension at this point which may only become evident with continued follow-up. Additionally, incidence of masked hypertension may differ between black and white populations [50].

In conclusion, when evaluating a detailed range of inflammatory mediators (individually or in clusters) in young healthy adults, we found independent relationships with ΔBP. Although black ethnicity strongly associated with ΔBP over time, associations between inflammatory mediators and ΔBP were evident in white adults only. These findings suggest that at this young age, the development of hypertension in black populations may not be driven by inflammation while, in the white population, subtle changes in inflammation may predict the early changes in BP.

## Access to data

The study methodology has been published [17], whereas the data dictionary, statistical analysis, protocol and deidentified individual participant data will be made available upon reasonable request to the corresponding author in agreement with all co-authors.

## Author contributions

**Simone H Crouch:** Conceptualisation, methodology, validation, formal analyses, investigation, data curation, writing - original draft, writing - review and editing, visualisation.

**Shani Botha-Le Roux:** Conceptualisation, methodology, validation, investigation, writing - review and editing.

**Christian Delles:** Validation, resources, writing - review and editing, funding acquisition.

**Lesley A Graham:** Validation, formal analyses, investigation, writing - review and editing.

**Aletta E Schutte:** Conceptualisation, methodology, validation, investigation, resources, writing - review and editing, funding acquisition.

## Sources of funding

The research funded in this manuscript is part of an ongoing research project financially supported by the 10.13039/501100001322South African Medical Research Council (SAMRC) with funds from the National Treasury under its Economic Competitiveness and Support Package; the 10.13039/100011971South African Research Chairs Initiative (SARChI) of the Department of Science and Technology and 10.13039/100011512National Research Foundation (NRF) of South Africa (GUN 86895; 10.13039/100006057SHC Grant Numbers: 123270); 10.13039/501100001322SAMRC with funds received from the South African National Department of Health, GlaxoSmithKline R&D (Africa Non-Communicable Disease Open Lab grant), the UK 10.13039/501100000265Medical Research Council and with funds from the UK Government’s Newton Fund; as well as corporate social investment grants from Pfizer (South Africa), 10.13039/100008349Boehringer-Ingelheim (South Africa), 10.13039/100004336Novartis (South Africa), the Medi Clinic Hospital Group (South Africa) and in kind contributions of 10.13039/100004337Roche Diagnostics (South Africa). CD is also supported by the 10.13039/501100000274British Heart Foundation (10.13039/100015258Centre of Research Excellence Awards RE/13/5/30177 and RE/18/6/34217).

## Disclosures

Any opinion, findings, and conclusions or recommendations expressed in this material are those of the authors, and therefore, the NRF does not accept any liability in this regard.

The authors report no conflict of Interest.

## References

[1] Barrows I.R., Ramezani A., Raj D.S. (2019). Inflammation, immunity, and oxidative stress in hypertension—partners in crime?. Adv. Chron. Kidney Dis..

[2] Stanaway J.D., Afshin A., Gakidou E. (2018). Global, regional, and national comparative risk assessment of 84 behavioural, environmental and occupational, and metabolic risks or clusters of risks for 195 countries and territories, 1990–2017: a systematic analysis for the Global Burden of Disease Study 2017. Lancet.

[3] Li J.-J., Fang C.-H., Hui R.-T. (2005). Is hypertension an inflammatory disease?. Med. Hypotheses.

[4] I Idris-Khodja N., Mian M.O.R., Paradis P., Schiffrin E.L. (2014). Dual opposing roles of adaptive immunity in hypertension. Eur. Heart J..

[5] Mian M.O.R., Paradis P., Schiffrin E.L. (2014). Innate immunity in hypertension. Curr. Hypertens. Rep..

[6] Harrison D.G., Guzik T.J., Lob H.E. (2011). Inflammation, immunity, and hypertension. Hypertension.

[7] Schiffrin E.L. (2014). Immune mechanisms in hypertension and vascular injury. Clin. Sci..

[8] Harrison D.G., Gongora M.C. (2009). Oxidative stress and hypertension. Med. Clin..

[9] Stumpf C., John S., Jukic J. (2005). Enhanced levels of platelet P-selectin and circulating cytokines in young patients with mild arterial hypertension. J. Hypertens..

[10] Sesso H.D., Buring J.E., Rifai N., Blake G.J., Gaziano J.M., Ridker P.M. (2003). C-reactive protein and the risk of developing hypertension. J. Am. Med. Assoc..

[11] Olsen M.H., Angell S.Y., Asma S. (2016). A call to action and a lifecourse strategy to address the global burden of raised blood pressure on current and future generations: the Lancet Commission on hypertension. Lancet.

[12] Pauletto P., Rattazzi M. (2006). Inflammation and hypertension: the search for a link. Nephrol. Dial. Transplant..

[13] Tziakas D.N., Chalikias G.K., Kaski J.C. (2007). Inflammatory and anti-inflammatory variable clusters and risk prediction in acute coronary syndrome patients: a factor analysis approach. Atherosclerosis.

[14] Crouch S.H., Botha-Le Roux S., Delles C., Graham L.A., Schutte A.E. (2020). Distinct inflammatory mediator patterns in young black and white adults: the African-predict study. Cytokine.

[15] Miller M., Cappuccio F. (2007). Ethnicity and inflammatory pathways-implications for vascular disease, vascular risk and therapeutic intervention. Curr. Med. Chem..

[16] Minor D.S., Wofford M.R., Jones D.W. (2008). Racial and ethnic differences in hypertension. Curr. Atherosclerosis Rep..

[17] Schutte A.E., Gona P.N., Delles C. (2019). The african prospective study on the early detection and identification of cardiovascular disease and hypertension (African-PREDICT): design, recruitment and initial examination. Eur J Prev Cardiol.

[18] Patro B.K., Jeyashree K., Gupta P.K. (2012). Kuppuswamy's socioeconomic status scale 2010—the need for periodic revision. Indian J. Pediatr..

[19] Williams B., Mancia G., Spiering W. (2018). 2018 ESC/ESH Guidelines for the management of arterial hypertension. Eur. Heart J..

[20] van Deventer H.E., George J.A., Paiker J.E., Becker P.J., Katz I.J. (2008). Estimating glomerular filtration rate in black South Africans by use of the modification of diet in renal disease and Cockcroft-Gault equations. Clin. Chem..

[21] Kilic T., Ural D., Ural E. (2006). Relation between proinflammatory to anti-inflammatory cytokine ratios and long-term prognosis in patients with non-ST elevation acute coronary syndrome. Heart.

[22] Gogos C.A., Drosou E., Bassaris H.P., Skoutelis A. (2000). Pro-versus anti-inflammatory cytokine profile in patients with severe sepsis: a marker for prognosis and future therapeutic options. J. Infect. Dis..

[23] Faul F., Erdfelder E., Lang A.-G., Buchner A.G. (2007). ∗ Power 3: a flexible statistical power analysis program for the social, behavioral, and biomedical sciences. Behav. Res. Methods.

[24] Simons K.H., de Jong A., Jukema J.W., de Vries M.R., Arens R., Quax P.H.A. (2019). T cell co-stimulation and co-inhibition in cardiovascular disease: a double-edged sword. Nat. Rev. Cardiol..

[25] Caillon A., Schiffrin E.L. (2016). Role of inflammation and immunity in hypertension: recent epidemiological, laboratory, and clinical evidence. Curr. Hypertens. Rep..

[26] Lande M.B., Pearson T.A., Vermilion R.P., Auinger P., Fernandez I.D. (2008). Elevated blood pressure, race/ethnicity, and C-reactive protein levels in children and adolescents. Pediatrics.

[27] Garanty-Bogacka B., Syrenicz M., Syrenicz A., Gebala A., Lulka D., Walczak M. (2005). Serum markers of inflammation and endothelial activation in children with obesity-related hypertension. Neuroendocrinol. Lett..

[28] Syrenicz A., Garanty-Bogacka B., Syrenicz M., Gebala A., Dawid G., Walczak M. (2006). Relation of low-grade inflammation and endothelial activation to blood pressure in obese children and adolescents. Neuroendocrinol. Lett..

[29] Devallière J., Charreau B. (2011). The adaptor Lnk (SH2B3): an emerging regulator in vascular cells and a link between immune and inflammatory signaling. Biochem. Pharmacol..

[30] Ye J., Que B., Huang Y. (2019). Interleukin-12p35 knockout promotes macrophage differentiation, aggravates vascular dysfunction, and elevates blood pressure in angiotensin II-infused mice. Cardiovasc. Res..

[31] McMaster W.G., Kirabo A., Madhur M.S., Harrison D.G. (2015). Inflammation, immunity, and hypertensive end-organ damage. Circ. Res..

[32] Stumpf C., Auer C., Yilmaz A. (2011). Serum levels of the Th1 chemoattractant interferon-gamma-inducible protein (IP) 10 are elevated in patients with essential hypertension. Hypertens. Res..

[33] Fitzgerald K.A., O'Neill L.A., Gearing A.J., Callard R.E. (2001). The Cytokine Factsbook and Webfacts.

[34] Li J.-J., Chen J.-L. (2005). Inflammation may be a bridge connecting hypertension and atherosclerosis. Med. Hypotheses.

[35] Mwantembe O., Gaillard M.-C., Barkhuizen M. (2001). Ethnic differences in allelic associations of the interleukin-1 gene cluster in South African patients with inflammatory bowel disease (IBD) and in control individuals. Immunogenetics.

[36] Schutte A.E., Myburgh A., Olsen M.H., Eugen-Olsen J., Schutte R. (2012). Exploring soluble urokinase plasminogen activator receptor and its relationship with arterial stiffness in a bi-ethnic population: the SAfrEIC-study. Thromb. Res..

[37] Selassie A., Wagner C.S., Laken M.L., Ferguson M.L., Ferdinand K.C., Egan B.M. (2011). Progression is accelerated from prehypertension to hypertension in blacks. Hypertension.

[38] Tanaka M. (2020). Improving obesity and blood pressure. Hypertens. Res..

[39] Yang M.H., Kang S.Y., Lee J.A., Kim Y.S., Sung E.J., Lee K.-Y., Kim J.-S., Oh H.J., Kang H.C., Lee S.Y. (2017). The effect of lifestyle changes on blood pressure control among hypertensive patients. Korean J Fam Med.

[40] Bochud M., Staessen J.A., Maillard M. (2009). Ethnic differences in proximal and distal tubular sodium reabsorption are heritable in black and white populations. J. Hypertens..

[41] Schutte A.E., Kruger R., Gafane-Matemane L.F., Breet Y., Strauss-Kruger M., Cruickshank J.K. (2020). Ethnicity and arterial stiffness. Arterioscler. Thromb. Vasc. Biol..

[42] Schutte A., Huisman H., Van Rooyen J. (2008). Should obesity be blamed for the high prevalence rates of hypertension in black South African women?. J. Hum. Hypertens..

[43] Huisman H.W., Schutte A.E., Schutte R. (2013). Exploring the link between cardiovascular reactivity and end-organ damage in African and Caucasian men: the SABPA study. Am. J. Hypertens..

[44] Drazner M.H., Dries D.L., Peshock R.M. (2005). Left ventricular hypertrophy is more prevalent in blacks than whites in the general population: the Dallas Heart Study. Hypertension.

[45] Schutte A., Botha S., Fourie C. (2017). Recent advances in understanding hypertension development in sub-Saharan Africa. J. Hum. Hypertens..

[46] Touyz R.M., Delles C. (2019). Textbook of Vascular Medicine.

[47] Terentes-Printzios D., Vlachopoulos C., Xaplanteris P. (2017). Cardiovascular risk factors accelerate progression of vascular aging in the general population: results from the CRAVE study (Cardiovascular Risk Factors Affecting Vascular Age). Hypertension.

[48] McDade T.W., Hoke M., Borja J.B., Adair L.S., Kuzawa C. (2013). Do environments in infancy moderate the association between stress and inflammation in adulthood? Initial evidence from a birth cohort in the Philippines. Brain Behav. Immun..

[49] Oparil S., Acelajado M.C., Bakris G.L., Berlowitz D.R., Cífková R., Dominiczak A.F., Grassi G., Jordan J., Poulter N.R., Rodgers A., Whelton P.K. (2018). Hypertension. Nat Rev Dis Primers.

[50] Thompson J.E., Smith W., Ware L.J. (2016). Masked hypertension and its associated cardiovascular risk in young individuals: the African-PREDICT study. Hypertens. Res..

