# Supplementary material for: Inflammation and hypertension development: A longitudinal analysis of the African-PREDICT study
Source: Int J Cardiol Hypertens. 2020 Nov 21;7:100067. doi: 10.1016/j.ijchy.2020.100067 (PMC7768897; doi:10.1016/j.ijchy.2020.100067)
Supplement: Multimedia component 1 [file mmc1.pdf]

Table S1. Characteristics of black and white individuals at baseline.

	**Black (n=202)**	**White (n=156)**	*p*
Age, years	24.4 ± 3.26	26.2 ± 2.75	**<0.001**
Men, n (%)	82 (40.6)	73 (46.8)	0.24
**Socio-economic Status**			
Low, n (%)	129 (63.9)	10 (6.4)	**<0.001**
Middle, n (%)	51 (25.2)	29 (18.6)	
High, n (%)	22 (10.9)	117 (75.0)	
**Body Composition**			
Body mass index (kg/m^2)	24.5 ± 5.45	26.5 ± 5.55	**0.001**
Waist circumference (cm)	76.9 ± 11.3	84.1 ±14.7	**<0.001**
**Office BP (mmHg)**			
SBP	118 ± 12.4	118 ± 12.3	0.66
DBP	79.7 ± 8.90	78.4 ± 8.40	0.18
**Ambulatory BP (mmHg)**			
24h SBP	115 ± 9.01	119 ± 10.1	**<0.001**
24h DBP	68.4 ± 5.86	69.7 ± 6.26	0.060
Daytime SBP	119 ± 9.43	124 ± 10.7	**<0.001**
Daytime DBP	72.8 ± 6.44	74.7 ± 10.7	**0.009**
Nighttime SBP	107 ± 10.3	109 ± 10.6	**0.035**
Nighttime DBP	60.1 ± 6.94	59.8 ± 6.54	0.81
Hypertensive, n (%)	34 (16.8)	39 (25.0)	**0.057**
**Inflammatory Markers**			
Pro-Inflammatory			
CRP (pg/mL)	1.24 (0.15; 9.41)	1.02 (0.12; 10.6)	0.18
Fractalkine (pg/mL)	27.6 (8.28; 72.5)	27.0 (11.1; 73.5)	0.74
INF-γ (pg/mL)	6.62 (1.61; 19.7)	7.33 (1.44; 21.0)	0.24
IL-1β (pg/mL)	0.94 (0.15; 3.79)	1.06 (0.27; 4.02)	0.23
IL-2 (pg/mL)	0.80 (0.12; 3.88)	0.79 (0.15; 3.96)	0.92
IL-7 (pg/mL)	5.62 (1.51; 17.3)	5.41 (0.86; 19.2)	0.68
IL-8 (pg/mL)	1.66 (0.45; 5.53)	1.95 (0.50; 6.99)	0.060
IL-12 (pg/mL)	1.76 (0.32; 6.47)	1.84 (0.45; 5.89)	0.64
IL-17 A (pg/mL)	3.19 (0.63; 12.9)	3.27 (0.64; 12.1)	0.80
IL-23 (pg/mL)	111 (13.5; 575)	134 (12.9; 772)	0.13
ITAC (pg/mL)	4.63 (1.46; 16.9)	3.24 (1.40; 8.63)	**<0.001**
MIP-*1α* (pg/mL)	8.75 (2.60; 22.9)	10.5 (3.47; 26.4)	**0.019**
MIP-*1β* (pg/mL)	6.92 (2.30; 14.9)	6.92 (3.00; 15.8)	1.00
MIP-3α (pg/mL)	2.26 (0.56; 8.68)	1.76 (0.57; 4.85)	**0.005**
TNF-α (pg/mL)	1.62 (0.39; 4.94)	1.72 (0.55; 5.20)	0.44
Anti-Inflammatory			
IL-4 (pg/mL)	43.4 (8.99; 153)	43.1 (9.28; 144)	0.94
IL-5 (pg/mL)	0.89 (0.19; 3.71)	1.04 (0.27; 3.88)	0.10
IL-10 (pg/mL)	4.34 (0.81; 17.4)	5.45 (0.94; 22.1)	**0.023**
IL-13 (pg/mL)	3.60 (0.54; 20.7)	5.51 (0.78; 31.0)	**<0.001**
Pro- and Anti-Inflammatory			
GM-CSF (pg/mL)	6.92 (1.35; 31.0)	8.12 (1.27; 30.9)	0.15

IL-6 (pg/mL)	1.73 (0.23; 8.60)	2.42 (0.28; 12.0)	**0.005**
IL-21 (pg/mL)	1.39 (0.23; 5.69)	1.35 (0.27; 4.82)	0.79
Pro-to-Anti Inflammatory Ratios			
IL-6 to IL-10	0.25 (0.04; 1.65)	0.15 (0.02; 1.23)	**<0.001**
IL-1β to IL-10	0.22 (0.08; 0.85)	0.19 (0.05; 0.73)	0.11
TNF-α to IL-10	0.38 (0.17; 1.03)	0.32 (0.13; 1.14)	**0.002**
CRP to IL-10	0.29 (0.02; 3.00)	0.19 (0.01; 3.54)	**0.011**
MIP-*1α* to IL-10	2.04 (0.72; 6.48)	1.84 (0.57; 6.50)	0.21
ITAC to IL-4	0.11 (0.02; 0.85)	0.08 (0.02; 0.41)	**0.001**
ITAC to IL- 5	5.42 (1.11; 39.0)	3.12 (0.76; 10.1)	**<0.001**
ITAC to IL-10	1.09 (0.26; 8.86)	0.60 (0.17; 2.88)	**<0.001**
ITAC to IL-13	1.30 (0.22; 9.88)	0.59 (0.09; 4.19)	**<0.001**

Fractalkine, Granulocyte-macrophage colony-stimulating factor (GM-CSF), Interferon gamma (IFN-γ), Interleukin 1 beta (IL-1β), Interleukin 2 (IL-2), Interleukin 4 (IL-4), Interleukin 5 (IL-5), Interleukin 6 (IL-6), Interleukin 7 (IL-7), Interleukin 8 (IL-8), Interleukin 10 (IL-10), Interleukin 12 (IL-12), Interleukin 13 (IL-13), Interleukin 17A (IL-17A), Interleukin 21 (IL-21), Interleukin 23 (IL-23), Interferon-inducible T-cell alpha chemoattractant (ITAC), Macrophage inflammatory protein *1-alpha* (MIP-1α), Macrophage inflammatory protein *1-beta* (MIP-1β), Macrophage inflammatory protein 3-*alpha* (MIP-3α) and Tumour Necrosis Factor Alpha (TNFα).
