# Supplementary material for: Inflammation and hypertension development: A longitudinal analysis of the African-PREDICT study
Source: Int J Cardiol Hypertens. 2020 Nov 21;7:100067. doi: 10.1016/j.ijchy.2020.100067 (PMC7768897; doi:10.1016/j.ijchy.2020.100067)
Supplement: Multimedia component 2 [file mmc2.pdf]

Table S2. Inflammatory mediator factor scores in the total population.

	**Factor 1**	**Factor 2**	**Factor 3**	**Factor 4**
CRP				-0.718
Fractalkine	0.636			
INF-γ	0.804			
IL-1β			0.609	
IL-2			0.819	
IL-7	0.698			
IL-8		0.716		
IL-12	0.698			
IL-17 A	0.776			
IL-23	0.629			
ITAC	0.510			
MIP-1α	0.571			
MIP-1β	0.687			
MIP-3α			0.759	
TNF-α	0.684			
IL-4	0.778			
IL-5			0.652	
IL-10	0.631			
IL-13		0.855		
IL-6		0.814		
IL-21			0.554	
GM-CSF	0.782			
**Eigenvalue**	8.35	2.61	3.36	-
**Cumulative %**	64.2	86.9	67.2	-
