# Supplementary material for: Inflammation and hypertension development: A longitudinal analysis of the African-PREDICT study
Source: Int J Cardiol Hypertens. 2020 Nov 21;7:100067. doi: 10.1016/j.ijchy.2020.100067 (PMC7768897; doi:10.1016/j.ijchy.2020.100067)
Supplement: Multimedia component 3 [file mmc3.pdf]

Table S3. Inflammatory mediator factor scores in the white population.

	Factor 1	Factor 2	Factor 3	Factor 4
CRP			0.347	
Fractalkine				0.711
INF-γ	0.831			
IL-1β			0.685	
IL-2			0.813	
IL-7	0.752			
IL-8		0.824		
IL-12	0.735			
IL-17 A	0.819			
IL-23	0.767			
ITAC				0.805
MIP-1α	0.548			
MIP-1β	0.754			
MIP-3α			0.691	
TNF-α	0.580			
IL-4	0.773			
IL-5		0.604		
IL-10	0.614			
IL-13		0.871		
IL-6		0.834		
IL-21	0.492			
GM-CSF	0.821			
**Eigenvalue**	8.37	3.23	2.23	1.46
**Cumulative %**	69.7	80.8	55.88	73.1
