# Supplementary material for: Inflammation and hypertension development: A longitudinal analysis of the African-PREDICT study
Source: Int J Cardiol Hypertens. 2020 Nov 21;7:100067. doi: 10.1016/j.ijchy.2020.100067 (PMC7768897; doi:10.1016/j.ijchy.2020.100067)
Supplement: Multimedia component 4 [file mmc4.pdf]

Table S4. Inflammatory mediator factor scores in the black population.

	**Factor 1**	**Factor 2**	**Factor 3**	**Factor 4**
CRP				-0.600
Fractalkine	0.636			
INF-γ	0.785			
IL-1β			0.609	
IL-2			0.834	
IL-7	0.617			
IL-8		0.691		
IL-12	0.633			
IL-17 A	0.709			
IL-23	0.596			
ITAC	0.590			
MIP-1α	0.575			
MIP-1β	0.573			
MIP-3α			0.725	
TNF-α	0.652			
IL-4	0.698			
IL-5			0.635	
IL-10	0.582			
IL-13		0.585		
IL-6		0.814		
IL-21			0.602	
GM-CSF	0.752			
**Eigenvalue**	8.41	2.56	3.39	---
**Cumulative %**	64.8	85.4	67.8	---
