# Supplementary material for: Inflammation and hypertension development: A longitudinal analysis of the African-PREDICT study
Source: Int J Cardiol Hypertens. 2020 Nov 21;7:100067. doi: 10.1016/j.ijchy.2020.100067 (PMC7768897; doi:10.1016/j.ijchy.2020.100067)
Supplement: Multimedia component 5 [file mmc5.pdf]

Table S5. Multivariable adjusted forward stepwise regression analyses to show the relationship between percentage change in blood pressure quartiles and clusters of inflammatory mediators in the white group.

Percentage change in 24hr SBP				
**White (n=156)**	**Q1**	**Q2**	**Q3**	**Q4**
Factor 1	---	---	---	---
Factor 2	---	---	β=-0.343<br>(-0.132; -0.101)<br>p=0.020	---
Factor 3	---	β=0.412<br>(0.016; 0.180)<br>p=0.021	---	---
Factor 4	---	---	---	---
**Percentage change in 24hr DBP**				
**White (n=156)**	**Q1**	**Q2**	**Q3**	**Q4**
Factor 1	---	---	---	---
Factor 2	---	---	---	---
Factor 3	---	---	---	---
Factor 4	---	---	---	---

Findings presented as β (95%CI).

Adjusted for: age, sex, socio-economic status, waist circumference, total cholesterol, glucose, gamma glutamyltransferase, cotinine, estimated glomerular filtration rate and activity energy expenditure.
