# Supplementary material for: Inflammation and hypertension development: A longitudinal analysis of the African-PREDICT study
Source: Int J Cardiol Hypertens. 2020 Nov 21;7:100067. doi: 10.1016/j.ijchy.2020.100067 (PMC7768897; doi:10.1016/j.ijchy.2020.100067)
Supplement: Multimedia component 6 [file mmc6.pdf]

Table S6. Multivariable adjusted forward stepwise regression analyses in the black and white group to show the relationship between percentage change in blood pressure and inflammatory mediators.

	Percentage change in					
**White**	**24hr SBP (n=156)**	**24hr DBP (n=156)**	**Daytime SBP (n=156)**	**Daytime DBP (n=155)**	**Nighttime SBP (n=154)**	**Nighttime DBP (n=154)**
Pro-Inflammatory						
CRP (mg/L)	---	---	---	---	---	---
Fractalkine (pg/mL)	---	---	---	---	---	---
IFN-γ (pg/mL)	---	---	R^2=0.041 β=0.220 (0.054; 0.390) P=0.010	---	---	---
IL-1β (pg/mL)	---	---	---	---	---	---
IL-2 (pg/mL)	---	---	---	---	---	---
IL-7 (pg/mL)	R^2=0.045 β=0.229 (0.055; 0.345) P=0.007	R^2=0.029 β=0.191 (0.021; 0.315) P=0.025	R^2=0.064 β=0.266 (0.096; 0.405) P=0.002	R^2=0.032 β=0.197 (0.027; 0.335) P=0.021	---	---
IL-8 (pg/mL)	---	R^2=0.029 β=0.189 (0.021; 0.344) P=0.027	R^2=0.028 β=0.190 (0.024; 0.369) P=0.026	R^2=0.037 β=0.211 (0.044; 0.380) P=0.014	---	---
IL-12 (pg/mL)	R^2=0.040 β=0.217 (0.048; 0.363) P=0.011	---	R^2=0.055 β=0.250 (0.086; 0.422) P=0.003	R^2=0.031 β=0.194 (0.026; 0.359) P=0.024	---	---
IL-17 A (pg/mL)	R^2=0.023 β=0.174 (0.006; 0.325) P=0.042	---	R^2=0.052 β=0.243 (0.080; 0.418) P=0.004	$R^2$0.032 β=0.198 (0.030; 0.364) P=0.021	---	---
IL-23 (pg/mL)	R^2=0.052 β=0.244 (0.068; 0.353)	R^2=0.029 β=0.172 (0.003; 0.294)	R^2=0.054 β=0.247 (0.076; 0.381)	R^2=0.023 β=0.174 (0.006; 0.309)	---	---

	P=0.004	P=0.045	P=0.004	P=0.042		
ITAC (pg/mL)	---	---	---	---	---	---
MIP-1α (pg/mL)	R^2=0.024<br>β=0.176<br>(0.008; 0.319)<br>P=0.040	R^2=0.028<br>β=0.186<br>(0.018; 0.330)<br>P=0.029	R^2=0.029<br>β=0.190<br>(0.022; 0.356)<br>P=0.026	---	---	---
MIP-1β (pg/mL)	R^2=0.024<br>β=0.177<br>(0.008; 0.321)<br>P=0.039	---	R^2=0.034<br>β=0.202<br>(0.036; 0.369)<br>P=0.018	---	---	---
MIP-3α (pg/mL)	---	---	---	---	---	---
TNF-α (pg/mL)	---	---	---	---	---	---
Anti-Inflammatory						
IL-4 (pg/mL)	R^2=0.023<br>β=0.175<br>(0.007; 0.354)<br>P=0.041	---	R^2=0.045<br>β=0.229<br>(0.070; 0.438)<br>P=0.007	---	---	---
IL-5 (pg/mL)	---	---	---	---	---	---
IL-10 (pg/mL)	R^2=0.025<br>β=0.182<br>(0.014; 0.330)<br>P=0.033	---	R^2=0.033<br>β=0.199<br>(0.033; 0.372)<br>P=0.020	R^2=0.021<br>β=0.169<br>(0.000; 0.334)<br>P=0.050	---	---
IL-13 (pg/mL)	R^2=0.044<br>β=0.226<br>(0.058; 0.377)<br>P=0.008	R^2=0.042<br>β=0.222<br>(0.054; 0.374)<br>P=0.009	R^2=0.055<br>β=0.249<br>(0.087; 0.427)<br>P=0.003	R^2=0.044<br>β=0.226<br>(0.060; 0.395)<br>P=0.008	---	R^2=0.027<br>β=0.185<br>(0.015; 0.310)<br>P=0.031
Pro- and Anti- Inflammatory						
IL-6 (pg/mL)	R^2=0.031<br>β=0.196<br>(0.028; 0.348)<br>P=0.022	R^2=0.031<br>β=0.195<br>(0.027; 0.349)<br>P=0.022	R^2=0.046<br>β=0.230<br>(0.066; 0.408)<br>P=0.007	R^2=0.035<br>β=0.206<br>(0.039; 0.375)<br>P=0.016	---	---
IL-21 (pg/mL)	R^2=0.041<br>β=0.220<br>(0.048; 0.343)	---	R^2=0.063<br>β=0.265<br>(0.098; 0.410)	R^2=0.026<br>β=0.182<br>(0.013; 0.326)	---	---

	P=0.010		P=0.002	P=0.034		
GM-CSF (pg/mL)	R^2=0.043<br>β=0.223<br>(0.058; 0.391)<br>P=0.009	---	R^2=0.052<br>β=0.242<br>(0.083; 0.439)<br>P=0.004	R^2-0.026<br>β=0.183<br>(0.015; 0.369)<br>P=0.033	---	---
Pro-to-Anti Inflammatory Ratios						---
IL-6/IL-10	---	---	---	---	---	---
IL-1β/IL-10	---	---	---	---	---	---
TNF-α/IL-10	---	---	---	---	---	---
CRP/IL-10	R^2=0.030<br>β=-0.192<br>(-0.371; -0.026)<br>P=0.024	---	R^2=0.037<br>β=-0.209<br>(-0.417; -0.048)<br>P=0.014	---	---	---
MIP-*1α*/IL-10	---	---	---	---	---	---
ITAC/IL-4	---	---	---	---	---	---
ITAC/IL- 5	---	---	---	---	---	---
ITAC/IL-10	---	---	---	---	---	---
ITAC/IL-13	---	---	R^2=0.026<br>β=-0.181<br>(-0.376; -0.015)<br>P=0.034	---	---	---
**Black**	**24hr SBP (n=198)**	**24hr DBP (n=200)**	**Daytime SBP (n=198)**	**Daytime DBP (n=200)**	**Nighttime SBP (n=186)**	**Nighttime DBP (n=187)**
All Pro-Inflammatory	---	---	---	---	---	---
All Anti-Inflammatory	---	---	---	---	---	---
All Pro- and Anti-Inflammatory	---	---	---	---	---	---
All Pro-to-Anti Inflammatory Ratios	---	---	---	---	---	---

Findings presented as β (95%CI).
Adjusted for: age, sex, socio-economic status, waist circumference, total cholesterol, glucose, gamma glutamyltransferase, cotinine, estimated glomerular filtration rate and activity energy expenditure.
