# Supplementary material for: Inflammation and hypertension development: A longitudinal analysis of the African-PREDICT study
Source: Int J Cardiol Hypertens. 2020 Nov 21;7:100067. doi: 10.1016/j.ijchy.2020.100067 (PMC7768897; doi:10.1016/j.ijchy.2020.100067)
Supplement: Multimedia component 7 [file mmc7.pdf]

Table S7. Hazard Ratio for the development of hypertension over 4.5 years

	**β**	**95%(CI)**	**p**
Factor 1	0.894	(0.704; 1.134)	0.36
Ethnicity (0, black / 1, white)	0.222	(0.112; 0.439)	**<0.001**
Waist Circumference (cm)	1.589	(1.255; 2.011)	**<0.001**
Glucose (mmol/L)	1.299	(0.938; 1.799)	0.12
Socio-economic Status	0.774	(0.567; 1.055)	0.11
Activity Energy Expenditure (kCal/day)	0.827	(0.640; 1.069)	0.15
Total Cholesterol (mmol/L)	0.827	(0.597; 1.147)	0.26
Estimated glomerular filtration rate (ml/min/1.73m^2)	0.874	(0.675; 1.132)	0.31
Cotinine (ng/ml)	1.211	(0.960; 1.528)	0.11
Gamma glutamyltransferase, (U/L)	1.053	(0.809; 1.369)	0.70
Sex (0, women / 1, men)	1.233	(0.773; 1.966)	0.38
Age (years)	1.225	(0.949; 1.581)	0.12
Factor 2	0.865	(0.687; 1.089)	0.22
Ethnicity (0, black / 1, white)	0.321	(0.176; 0.588)	**<0.001**
Waist Circumference (cm)	1.409	(1.133; 1.752)	**0.002**
Glucose (mmol/L)	1.180	(0.853; 1.631)	0.32
Socio-economic Status	0.777	(0.576; 1.047)	0.098
Activity Energy Expenditure (kCal/day)	0.901	(0.709; 1.144)	0.39
Total Cholesterol (mmol/L)	0.882	(0.637; 1.221)	0.45
Estimated glomerular filtration rate (ml/min/1.73m^2)	0.886	(0.692; 1.134)	0.34
Cotinine (ng/ml)	1.148	(0.918; 1.436)	0.23
Gamma glutamyltransferase, (U/L)	1.182	(0.937; 1.490)	0.16
Sex (0, women / 1, men)	1.151	(0.737; 1.796)	0.54
Age (years)	1.189	(0.940; 1.503)	0.15
Factor 3	0.778	(0.611; 0.990)	**0.042**
Ethnicity (0, black / 1, white)	0.225	(0.112; 0.454)	**<0.001**
Waist Circumference (cm)	1.565	(1.227; 1.995)	**<0.001**
Glucose (mmol/L)	1.461	(1.018; 2.098)	**0.040**
Socio-economic Status	0.769	(0.553; 1.069)	0.12
Activity Energy Expenditure (kCal/day)	0.854	(0.656; 1.112)	0.24
Total Cholesterol (mmol/L)	0.897	(0.640; 1.257)	0.53
Estimated glomerular filtration rate (ml/min/1.73m^2)	0.878	(0.672; 1.147)	0.34
Cotinine (ng/ml)	1.171	(0.929; 1.477)	0.18
Gamma glutamyltransferase, (U/L)	1.130	(0.872; 1.465)	0.36
Sex (0, women / 1, men)	1.058	(0.654; 1.711)	0.82
Age (years)	1.251	(0.966; 1.621)	0.090

Factor 1: Fractalkine, IFN-γ, IL-4, IL-7, IL-10, IL-12, IL-17A, IL-23, ITAC, MIP-1α, MIP-1β, TNF-α, GM-CSF

Factor 2: IL-6, IL-8, IL-13

Factor 3: IL-1β, IL-2, IL-5, IL-21, MIP-3α
